# A commentary on ‘BF.7 Omicron subvariant (BA.5.2.1.7) posing fears of a rise in COVID-19 cases again: a critical appraisal and salient counteracting strategies’

**DOI:** 10.1097/JS9.0000000000000766

**Published:** 2023-09-21

**Authors:** Andi Chen, Xiaohui Chen, Shishi Huang, Xiaochun Zheng

**Affiliations:** a Department of Anesthesiology, Shengli Clinical Medical College of Fujian Medical University, Fujian Provincial Hospital; b Department of Anesthesiology, Fujian Medical University Union Hospital; c Fujian Emergency Medical Center, Fujian Provincial Key Laboratory of Emergency Medicine, Fujian Provincial Key Laboratory of Critical Care Medicine, Fujian Provincial Co-Constructed Laboratory of “Belt and Road”, Fuzhou, People’s Republic of China

*Dear Editor,*

We read with great interest the article entitled ‘BF.7 Omicron subvariant (BA.5.2.1.7) posing fears of a rise in COVID-19 cases again: a critical appraisal and salient counteracting strategies’ published in the recent issue (2023) of the *International Journal of Surgery* ^1^. The authors expressed concern over the global circulation, high prevalence, and increased transmission power of the Omicron subvariant BF.7 (BA.5.2.1.7). They emphasized the need for preparedness at all levels of the organization and urged proactive measures to mitigate the high global health concerns and socioeconomic impact for current Omicron subvariant and future mutant or notable variation strains. Anesthesiologists who always perform endotracheal intubation on patients positive for the Omicron variant during surgeries should prioritize their attention to this issue.

As the epidemic progresses, surgery on Omicron-positive patients has become inevitable and poses a significant risk to anesthetists performing endotracheal intubation. Aerosols are the main mode of transmission of Omicron, which is described as ‘Patients can produce high concentrations of infectious respiratory aerosols by coughing, sneezing, talking or breathing’^2^. Among the aerosol-generating procedures, performing endotracheal intubation is especially hazardous. Therefore, it is crucial for anesthesiologists to be well protected against a standing Omicron epidemic.

Currently, there are three potential comprehensive protective measures available for anesthesiologists during endotracheal intubation procedures:

The first step is to strengthen the anesthesiologist’s own immune system and constitution. With the prevalence of COVID-19 in the world as a dangerous disease, many studies have given physical exercise interventions^3^. Undeniable evidence^4,5^ has been supporting the vital role of physical exercise in improving the immune system, body composition, and reducing the complications of COVID-19. The WHO reported that all adults should aim for 150–300 min of moderate-intensity physical activity per week or 75–150 min of vigorous-intensity physical activity per week, or an equivalent combination of moderate-intensity and vigorous-intensity physical activity^6^. We therefore call on anesthesiologists to exercise to improve fitness in accordance with the WHO report. In addition, as the new coronavirus continues to evolve, the development of vaccines can be the most prominent approach to prevent the virus from causing COVID-19^7^. We would like to reiterate our call for anesthesiologists to get the new booster vaccine in a timely manner.

Secondly, it is important for medical institutions to create guidelines for the airway management of Omicron patients and an infrastructure to provide sufficient personal protective equipment (PPE) for intubating teams. For example, the previous report mentioned we can use as a reference^8^; detailed content is as follows:

Guidelines for tracheal intubation: (1) rapid sequence intubation and avoidance of bag-mask ventilation, if possible; (2) use of video laryngoscopy to increase the distance from the patient’s airway and the chance of success during first attempt; (3) immediate inflation of the tracheal tube cuff and connection to the ventilatory circuit, thereby avoiding manual ventilation; (4) use of a high-efficiency particulate air filter placed on the expiratory limb of a ventilator, or between the tracheal tube and a self-inflating bag ventilator.

PPE for tracheal intubation: (1) N95 mask; (2) disposable waterproof gowns for intubating provider and airway assistant; (3) eye/face protection: welder-style face mask or surgical mask with face shield attached; (4) high-efficiency particulate air (HEPA) filter.

Thirdly, the recent focus on the critical setting, especially with the COVID-19 pandemic, has highlighted the need for minimizing contact and increasing robotic use. Robotics is a rising field in the context of health care, especially in endotracheal intubation^9^. We call on more institutions to work together to develop intubation robots to replace anesthesiologists in the endotracheal intubation of patients with more potent variants of toxins, which is also one of the main research directions of our team at present.

All in all, anesthesiologists performing endotracheal intubation are already unavoidably exposed to significant risks of Omicron epidemics, but as anesthesiologists, we have our own duties and responsibilities. We believe that based on the above aspects of the call and the improvements made in practice, we will be able to overcome the epidemic.

## Ethical approval

This article does not require any human or animal subjects to acquire such approval.

## Consent

This article does not require any human or animal subjects to acquire such approval.

## Sources of funding

No funding was received.

## Author contribution

X.Z.: conceptualized, reviewed, and edited; A.C., X.C., and S.H.: writing and making the draft and updating it. All authors have critically reviewed and approved the final draft and are responsible for the content and similarity index of the manuscript.

## Conflicts of interest disclosure

The authors declare no conflicts of interest.

## Research registration unique identifying number (UIN)

None.

## Guarantor

All authors.

## Data availability statement

This study did not have any data analysis.

## Provenance and peer review

Not commissioned, externally peer-reviewed.
